# Dose-response relationship between daily screen time and the risk of low back pain among children and adolescents: a meta-analysis of 57831 participants

**DOI:** 10.1265/ehpm.23-00177

**Published:** 2023-10-27

**Authors:** Cheng Yue, Guo Wenyao, Ya Xudong, Shao Shuang, Shao Zhuying, Zhu Yizheng, Zhou Linlin, Chen Jinxin, Wang Xingqi, Liu Yujia

**Affiliations:** 1 Department of Physical Education, Jiangsu Normal University, Xuzhou, Jiangsu Province, 221116, China; 2 Biomedical R&d Center, School of Life Science, Jiangsu Normal University, Xuzhou, Jiangsu Province, 221116, China; 3 Yancheng Xinyang Youth Development Service Center, Yancheng, Jiangsu Province, 224000, China

**Keywords:** Screen, Low back pain, Adolescent, Dose-response, Meta-analysis

## Abstract

**Background:**

The risk of low back pain (LBP) increases steeply during adolescence, and adolescents with LBP are more likely to have low back pain in their adult years. This study aimed to investigate the dose-response relationship between daily screen time and the risk of low back pain among children and adolescents.

**Methods:**

PubMed, the Cochrane Library, Embase, and Web of Science were searched to collect relevant studies on daily screen time and the risk of low back pain from the establishment of the database up to December 2022. Two investigators independently screened the literature, extracted data, and evaluated the risk of bias in the included studies. Stata16.0 was used to perform a dose-response meta-analysis and the methodological quality evaluation of the included studies.

**Results:**

The results of the meta-analysis showed that there is a positive correlation between daily computer time (OR = 1.32, 1.05–1.60), daily mobile phone time (OR = 1.32, 1.00–1.64), daily TV watching (OR = 1.07, 1.04–1.09) and the risk of low back pain, separately. The dose–response meta-analysis showed that there is a linear relationship between daily computer use and low back pain. The risk of low back pain increased by 8.2% for each 1-hour of daily computer use.

**Conclusions:**

Screen time is related to the risk of low back pain, and there is a linear relationship between daily computer use and the risk of low back pain. A number of strategic measures should be taken to prevent adolescents from developing severe low back pain.

## Graphical Abstract

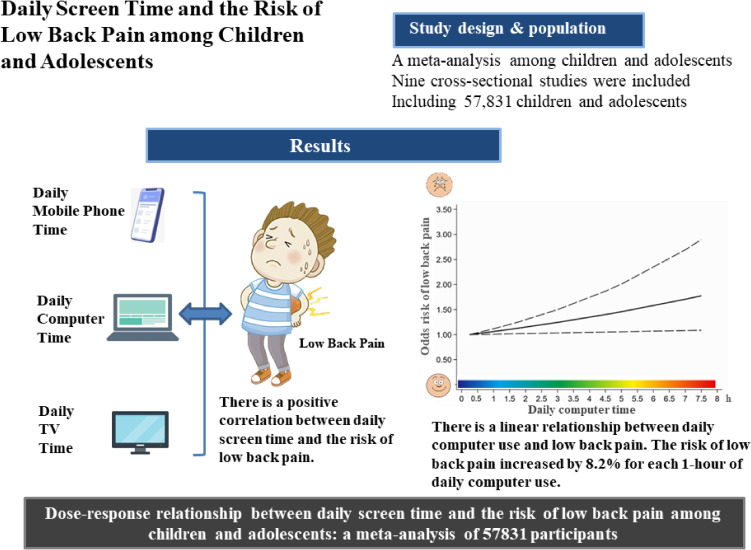

## Background

Low back pain (LBP), which is defined by the location of pain, typically occurs between the lower rib margins and the buttock creases [1]. It is commonly accompanied by lower extremity irradiating pain [2]. and does not have a clearly identifiable cause [3]. Globally, LBP is the leading cause of years lost to disability and the main contributor to the overall burden of disease [4]. It is common in the working population [2], and is a leading cause of job loss, reduced productivity, increased financial compensation, and hiring costs among workers [5–8]. Research has shown that the prevalence of LBP increases steeply during adolescence [9], and adolescents with LBP are more likely to have low back pain in their adult years [10]. Knowing its potential risk factors and implementing effective preventive measures among children and adolescents are urgently necessary.

Studies suggest that screen time may be a risk factor for LBP [11, 12]. In recent years, increasing screen time among teenagers has become a significant concern [13]. And COVID-19 will worsen this situation because approximately 80% of the world’s student population is enrolled in e-learning according to UNESCO statistics [14]. Previously, a study found that increases in screen time was associated with chronic back pain among European adolescents [15]. Meanwhile, a systematic review found that prolonged TV watching and computer/mobile use, and console playing time were significantly associated with LBP among children [16]. However, the dose-response relationship between screen time and LBP has not been explored in previous research.

Thus, we aimed to assess quantitatively the relationship between screen time and the risk of LBP by using a dose-response meta-analysis. Meanwhile, providing a theoretical background to help adolescents reasonably arrange their daily activities as well as improve prevention and treatment programs for LBP.

## Methods

### Literature search strategy

We followed the Preferred Reporting Items for Systematic Reviews and Meta-analyses (PRISMA) as the protocol for designing this review. Databases including PubMed, the Cochrane Library, Embase, Web of Science were searched for all studies investigating the association between daily screen time and the risk of LBP from their inception until December 21st, 2022. We used search terms including “back pain”, “screen”, “computer/PC/tablet” “smartphone/phone/mobile/cellphone”, “TV/television”. The search strategy is shown below: ((((((((((screen time[Title/Abstract]) OR (computer time[Title/Abstract])) OR (PC time[Title/Abstract])) OR (tablet time[Title/Abstract])) OR (smartphone time[Title/Abstract])) OR (phone time[Title/Abstract])) OR (mobile time[Title/Abstract])) OR (cellphone time[Title/Abstract])) OR (TV time[Title/Abstract])) OR (television time[Title/Abstract])) AND ((back pain[Title/Abstract]) OR (backache[Title/Abstract])). The papers’ references were also searched as supplements.

### Inclusion criteria

Studies had to meet the following criteria: (1) Cross-sectional or cohort studies; (2) Identified the participants’ source and age range (≤20 years old); (3) Reported the daily screen time; (4) The LBP prevalence was reported as an outcome variable or could be calculated; (5) The odds ratio (OR) and 95% confidence interval (CI) were provided or could be calculated between daily screen time and the prevalence of LBP.

### Study selection and data extraction

The studies that met the inclusion criteria were reviewed and extracted independently by two researchers. We reviewed the titles and abstracts, eliminated non-conforming studies, extracted the data, and cross-checked the data using the full text. Disagreements were resolved by discussion between two researchers. We extracted data content like follows: The name of the first author, year of publication, country, number of subjects, participants’ age and gender, the daily screen time acquisition methods, the risk of LBP, the stratification and median of daily screen time, the outcome cases and the total cases of the stratified analyses, adjusted OR value and 95% CI. For the studies that presented OR values based on gender or device, we extracted adjusted OR values, separately. As a result, certain studies appear twice in the figures of the results. E-mailed the corresponding author if any data were incomplete. The midpoint between the upper and lower limit was used as the median if the study did not include a median for stratified screen time. In the event that the stratification was an open interval, refer to other intervals.

### Study quality evaluation

Two investigators independently evaluated the quality of the included studies. This study used the American Institute for Health Care Research and Quality Scale (AHRQ) [17] to assess the cross-sectional studies. There are 11 evaluation criteria recommended. A “yes” score is one, while a “no” or “unclear” score is zero. Studies scoring 0–3 were defined as low quality, 4–7 as average quality, and 8–11 as high quality. Disagreements in scores between reviewers were resolved by discussion with a third party.

### Statistical analysis

Stata 16.0 software was used for the statistical analysis, Cochrane’s Q test was used to evaluate heterogeneity between the included studies, and I^2^ was used to calculate heterogeneity quantitation. Statistical heterogeneity between studies was low for P > 0.1 and I^2^ < 50%. The combined OR and 95% CI were calculated using a random effects model when I^2^ > 50%, and the fixed effects model was used and vice versa. In each study, a meta-analysis of daily screen time and the risk of LBP used the highest and lowest time stratification. Potential publication bias was assessed by Begg’s test and the asymmetry of funnel plots. For the further analysis of linear or nonlinear associations, the restrict cubic spline analysis method and generalized least-squares method were applied. P_Nonlinearity_ < 0.05 meant there was a nonlinear relationship and a nonlinear model was fitted. Otherwise, the linear model was fitted. Due to insufficient data on mobile devices and TV watching in the original studies, we only conducted a dose-response meta-analysis on the relationship between daily computer time and the risk of LBP.

## Results

### Study selection

**Fig. 1:**
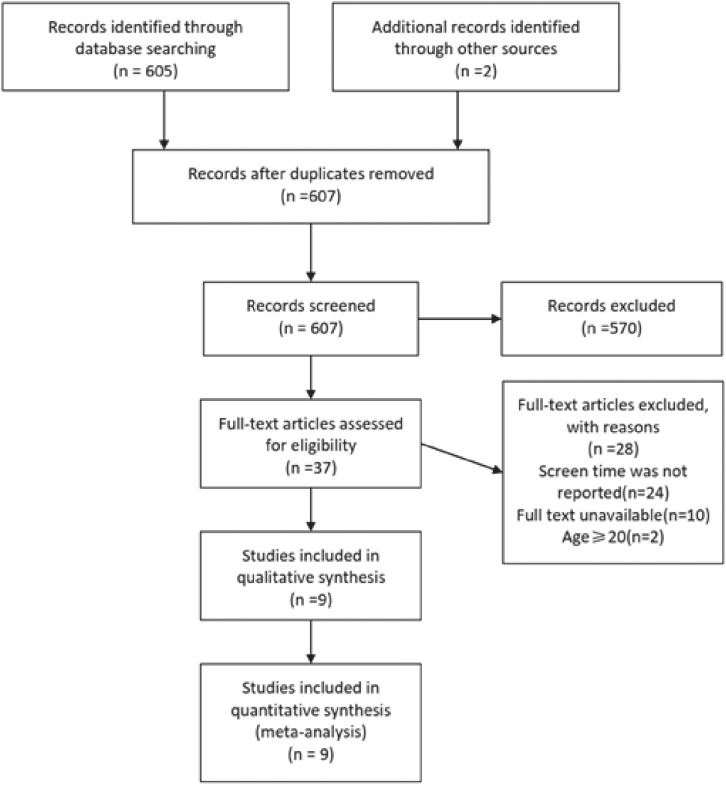
PRISMA flow diagram of the study selection process.

The study screening process and results were shown in Fig. 1. Using keywords related to daily screen time and LBP, 607 studies were found. Following the removal of duplicate studies, 37 studies were obtained by preliminary screening based on the titles and abstracts. After reading the full text, nine cross-sectional studies were ultimately included [18–26].

### Characteristics of the included studies

According to Table 1, nine cross-sectional studies investigated the relationship between daily screen time and LBP [18–26], including 99,311 participants. These studies were all included to examine the association between computer time and LBP [18–26]. Four studies were included to examine the association between phone time and LBP [18, 20, 24, 25], including 11,616 participants. Four studies were included to examine the association between TV time and LBP [18, 19, 21, 25], including 46,695 participants. Among the included studies, six studies were conducted in Europe, two studies were conducted in South America, and one study was conducted in Asia. All the included participants were between the ages of 11 and 19. According to the AHRQ scales, all included studies were of high or moderate quality (Table 2).

**Table 1:** Characteristics of researches included.

**Study**	**country**	**Participants**	**Cohort size**	**Sex**	**Age (years)**	**Data acquisition**	**Screen device reported**			**Covariates**	**Literature quality evaluation score**

							**computer**	**TV**	**mobile device**		
Hakala 2006	Finland	adolescents	6003	Male = 2665 Female = 3338	14∼18	Self-report	✓	✓	✓	Age, sex, school success, timing of puberty, stress symptoms	8
Torsheim 2010	Denmark	adolescents from Nordic countries	31022	NM	11,13,15	Self-report	✓	✓		Country, age, socioeconomic status, depression, school stress, physical activity	7
Shan 2013	China	students	3016	Male = 1460 Female = 1556	15∼19	Self-report	✓		✓	Sex, grade, soreness after exercise, digital device use, sitting time after school and personal emotions	8
Brindova 2015	Slovak	students	8042	Male = 3,910 Female = 4,132	11∼15	Self-report	✓	✓		Age, sex	7
Rossi 2016	Finland	students	1637	Male = 772 Female = 865	14∼16	Self-report	✓			Age, sex, BMI, chronic diseases, smoking	8
Noll 2016	Brazil	students	1439	Male = 765 Female = 674	11∼16	Self-report	✓	✓		Age, sex	8
Silva 2017	Portugal	students	969	Male = 467 Female = 502	13∼19	Self-report	✓		✓	Age, sex, BMI, physical activity, sleep quality	7
Bento 2020	Brazil	students	1628	Male = 798 Female = 830	14∼18	Self-report	✓		✓	Sex, type/time of computer, daily use time/posture (mobile phone), daily use time (tablet), mental health problems	7
Joergensen 2021	Denmark	pre-adolescents	45,555	Male = 21,711 Female = 23,844	11∼12	Self-report	✓			Sex, age, family type, on parental education, household income, physical activity	7

NM = not mentioned.

**Table 2:** Quality assessment of included studies.

**Study**	**Item 1**	**Item 2**	**Item 3**	**Item 4**	**Item 5**	**Item 6**	**Item 7**	**Item 8**	**Item 9**	**Item 10**	**Item 11**	**Total score**
Hakala et al, 2006	✓	✓	✓	✓	✓	×	/	✓	✓	✓	/	8
Torsheim et al, 2010	✓	✓	✓	✓	✓	×	/	✓	/	✓	/	7
Shan et al, 2013	✓	✓	✓	✓	✓	×	✓	✓	/	✓	/	8
Brindova et al, 2015	✓	✓	✓	✓	✓	×	✓	×	/	✓	/	7
Rossi et al, 2016	✓	✓	✓	✓	✓	×	✓	✓	/	✓	/	8
Noll et al, 2016	✓	✓	✓	✓	✓	×	✓	✓	/	✓	/	8
Silva et al, 2017	✓	✓	✓	✓	✓	×	/	✓	/	✓	/	7
Bento et al, 2020	✓	✓	✓	✓	✓	×	✓	×	/	✓	/	7
Joergensen et al, 2021	✓	✓	✓	✓	✓	×	/	✓	/	✓	/	7

Table notes: ✓ means yes; × means no; / means unclear; Item 1: Define the source of information (survey, record review); Item 2: List inclusion and exclusion criteria for exposed and unexposed participants (cases and controls) or refer to previous publications; Item 3: Indicate time period used for identifying patients; Item 4: Indicate whether or not participants were consecutive if not population-based; Item 5: Indicate if evaluators of subjective components of study were masked to other aspects of the status of the participants; Item 6: Describe any assessments undertaken for quality assurance purposes (e.g., test/retest o primary outcome measurements); Item 7: Explain any patient exclusions from analysis; Item 8: Describe how confounding was assessed and/or controlled; Item 9: If applicable, explain how missing data were handled in the analysis; Item 10: Summarize patient response rates and completeness of data collection; Item 11: Clarify what follow-up, if any, was expected and the percentage of patients for which incomplete data or follow-up was obtained. A “yes” score is one, while a “no” or “unclear” score is zero. Studies scoring 0–3 were defined as low quality, 4–7 as average quality, and 8–11 as high quality.

### The results of meta-analysis

**Fig. 2:**
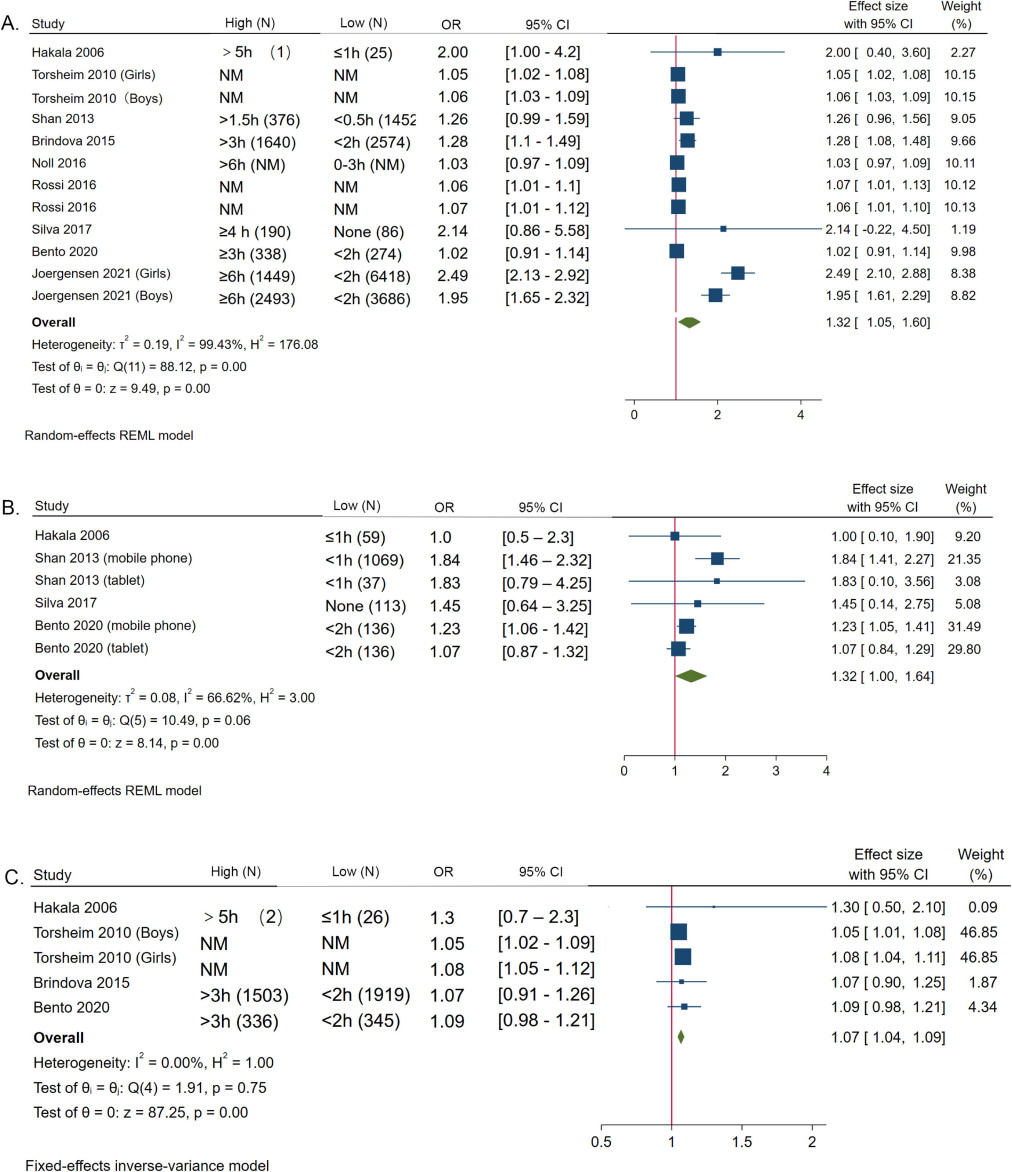
Association between daily screen time and the risk of LBP. A. Association between daily computer time and the risk of LBP. B. Association between daily mobile phone time and the risk of LBP. C. Association between daily TV watching and the risk of LBP.

The results of meta-analysis showed that compared with the shortest computer time, the risk of LBP was higher in participants who used computers for a longer time per day (OR = 1.32, 95% CI 1.05–1.60). The values of I^2^ = 99.43% and P < 0.001 represented high heterogeneity. The risk of LBP was higher in participants who used a mobile phone for a longer time per day (OR = 1.32, 95% CI 1.00–1.64). I^2^ = 66.62%, P < 0.001, meant moderate heterogeneity. The risk of LBP was higher in those who watched TV daily more (OR = 1.07, 95% CI 1.04–1.09), and the value of I^2^ = 0.00%, meant no heterogeneity. (Fig. 2.)

### Risk of publication bias

**Fig. 3:**
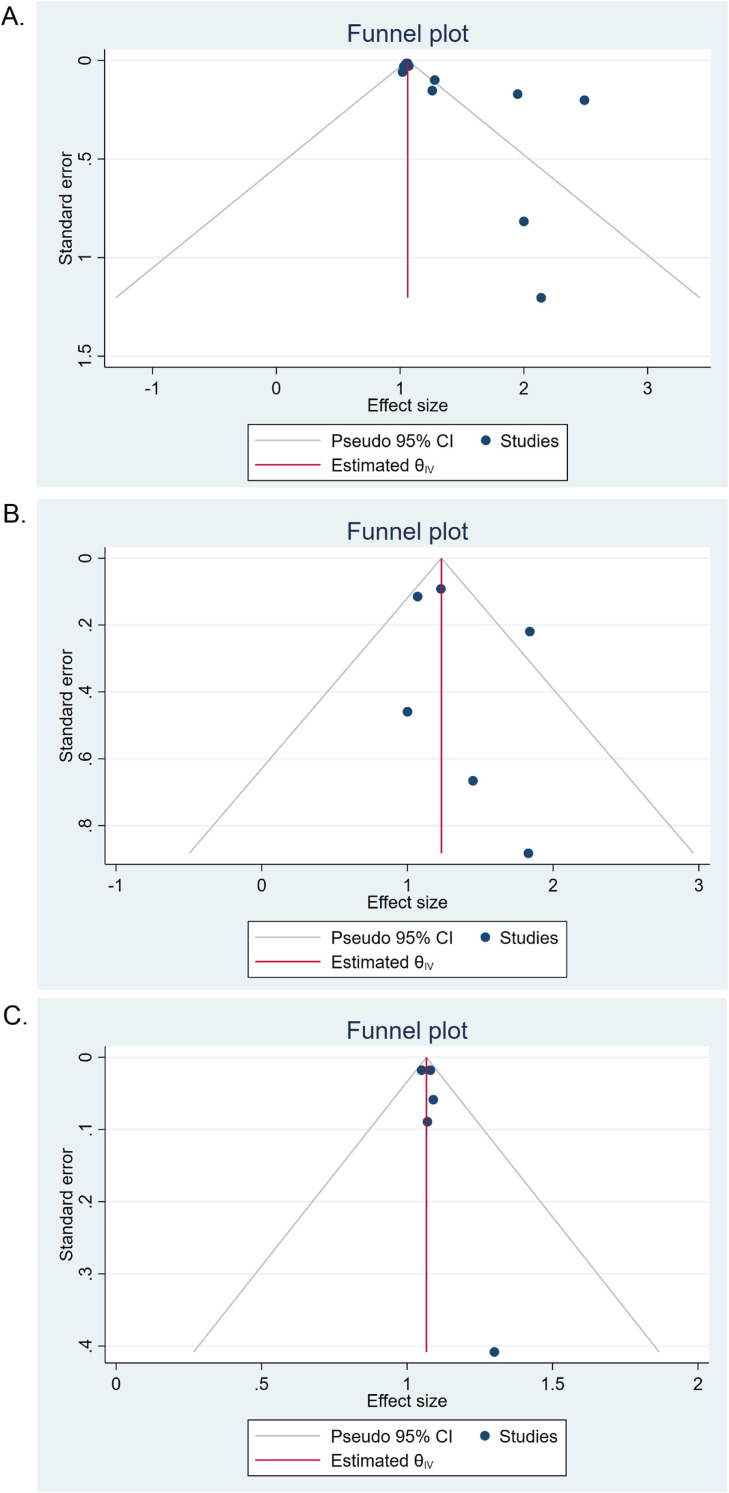
Bias funnel plot of the association between screen time and the risk of LBP. A. Bias funnel plot of the association between daily computer time and the risk of LBP. B. Bias funnel plot of the association between daily mobile phone time and the risk of LBP. C. Bias funnel plot of the association between daily TV watching and the risk of LBP.

Begg’s test and funnel plot were used to assess potential publication bias. The vertical line represents zero sizes. Each dot represented one study. The funnel plot of publication bias between daily screen time and the risk of LBP was asymmetric (Fig. 3), indicating the possibility of publication bias. For further analysis, the cut-and-fill method was used, and results showed that there was no significant difference between results after correction.

### The dose-response meta-analysis of daily computer time and the risk of LBP

**Fig. 4:**
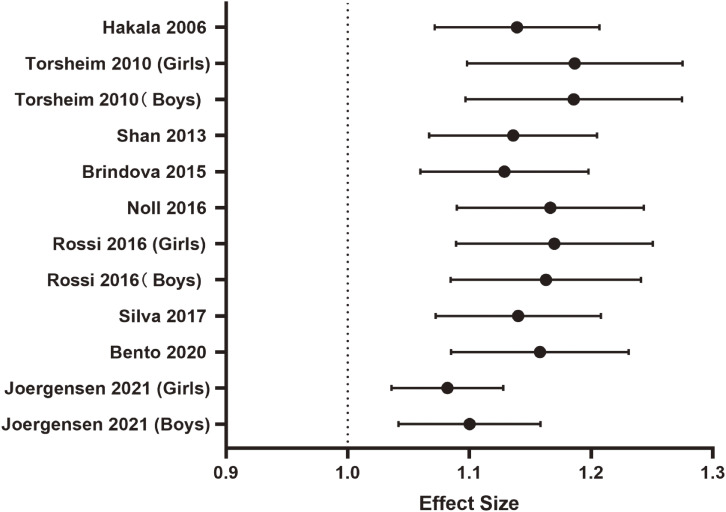
A sensitivity analysis of daily computer time and LBP. Each circle represents the estimated effect and 95% confidence interval when the study was excluded.

Considering the high heterogeneity in the relationship between computer time and LBP, we conducted a sensitivity analysis before exploring the dose-response relationship between the two variables. As shown in Fig. 4, the results of the sensitivity analysis were stable, we further investigated and discovered the dose-response relationship between the two variables.

**Fig. 5:**
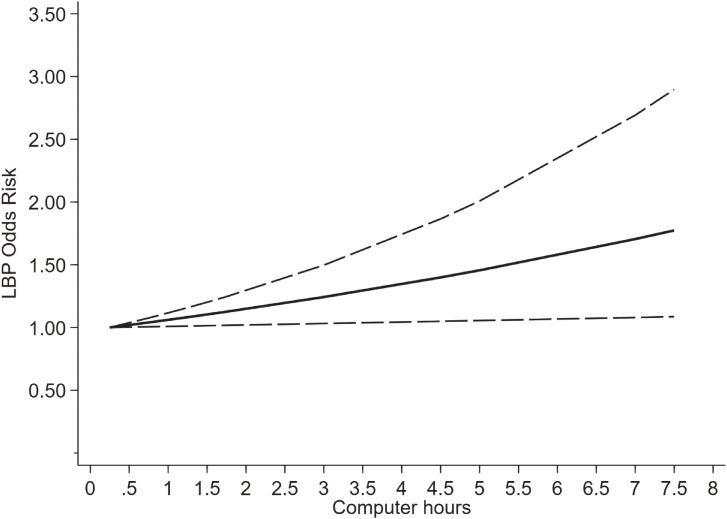
Dose-response relationship between daily computer time and the risk of LBP.

Torsheim et al. [19] didn’t report the number of sample and patients in each screen time subgroup, so that seven studies of six articles [18, 20–22, 24, 26] were finally included to interpret the dose-response relationship between daily computer time and the risk of LBP, including 57831 participants. We found a linear dose-response relationship between them (P_nonlinear_ > 0.05). For each 1-hour increase in daily computer time, the risk of LBP increases by 8.2%. (Fig. 5.)

## Discussion

This study examined the correlation between screen (mobile, TV, computer) time and LBP and whether there is a dose-response relationship. We assessed quantitatively the relationship between screen time and the risk of LBP by using a dose-response meta-analysis for the first time. We extracted and combined adjusted OR from nine cross-sectional research and found that screen time is positively correlated with the risk of LBP, and that there is a linear dose-response relationship between daily computer time and LBP. With electronic devices becoming more accessible and commonly abused, this study may help adolescents manage their screen time wisely.

Many studies have linked screen time to LBP among adolescents, and have found that screen time over a certain threshold per day was associated with LBP among children and adolescents. Hakala et al. [18] found that daily use of computers exceeding five hours seems to be a threshold for LBP among Finnish adolescents. AlShayhan et al. [27] found that spending more than ten hours on computer or tablet was significantly associated with LBP. While Bento et al. [25] found that TV use, tablet use, and cell phone use for more than three hours a day were associated with LBP. A cohort study of young adults concluded that the risk of LBP was 1.19 times higher among those who used mobile devices for 7 h or more a day [28]. Studies reported differing screen time thresholds associated with LBP may be related to the different time stratification. A systematic review found that there is a significant association between TV watching, computer/mobile use, console play time, and LBP [16], which confirmed the correlation between screen time and LBP. Our results are consistent with these results. We extracted and combined the adjusted OR from nine cross-sectional research to exclude the influence of confounders. We found that there is a positive correlation between daily computer use (OR = 1.32, 1.05–1.60), daily mobile use (OR = 1.32, 1.00–1.64), daily TV watching (OR = 1.07, 1.04–1.09) and the risk of LBP, separately. We found a linear relationship between daily computer use and LBP, with an 8.2% rise in LBP for every hour of computer use.

Heterogeneity was detected in the results about daily computer use, daily mobile use, and the risk of LBP. The participants from four included studies were from Northern Europe [18, 19, 23, 26], one study was from Portugal [24], two studies were from Brazil [22, 25], one study was from China [20], and one of the studies originated from Central Europe [21]. All the participants were students between the ages of 11 and 19. Different regions and ages may have variations in their education systems, stemming from cultural, and socioeconomic, which may be the sources of heterogeneity. In our study, we extracted adjusted OR to eliminate the influence of confounding factors. Thereby, the classification criteria of video time interval varied in different studies, which may also lead to heterogeneity. Importantly, heterogeneity may also be caused by different definitions of LBP and different stratifications of screen time in the original studies. Several studies defined LBP as occurring within the past month [25], while some defined it as occurring within the past year [25] or the half past year [18]. As a multidimensional experience, there is no gold standard to measure pain. The participants’ LBP frequency was mostly gathered from self-reported questionnaires, which were subject to memory bias resulting in heterogeneity. Joergensen et al. [26] constructed one stratification every two hours, while Bento et al. [25] used one hour as a stratification. The different stratifications of screen time in each original study may lead to the different risk of LBP per stratification in our study, which may be the most likely source of heterogeneity.

More and more studies suggest that the correlation between screen time and the risk of LBP may be due to ergonomic factors. Increasing numbers of people are spending long hours at their computers [29]. More ergonomic studies have been conducted on computer use than on TV or mobile phones. Pillastrini et al. [30] performed ergonomic interventions among video display terminal operators and their results showed that ergonomic adjustment could be reducing LBP symptoms. In that study, participants’ LBP symptom was relieved by adjusting chair and desk height, backrest inclination, screen height, inclination and orientation, mouse location, keyboard inclination, and location, which suggests that these factors may play a mediating role in computer use and LBP. Furthermore, several studies have found that the computer location (monitor not in front) [31], the absence of low back support during computer work [32], are all the factors that significantly associated with computer use and the risk of LBP. Emerson et al. [33] proposed a set of ideal ergonomic recommendations for computer workstations to reduce musculoskeletal pain and symptoms. In recent years, ergonomic advice on computer use has been more comprehensive, which may prevent and improve LBP. However, the patency rates of ergonomics of computer use that comply with recommendations is unclear. We tentatively put forward that reducing screen time may be an effective way to reduce the risk of LBP.

In addition to ergonomic factors, there are also several health-related factors that may be associated with screen time and LBP. We know that the physical health of adolescents has long been endangered by issues including obesity [34], sleep quality [35], and mental health [36]. Researchers also found a positive association between these factors and LBP [37–39]. In recent years, there has been a significant increase in screen time among adolescents, which may have adverse effects on their health. Research has found a significant correlation between screen time and obesity among children [40, 41]. Khan et al. [42] found that excessive screen time of any type was associated with sleep difficulties among adolescents. Paulich et al. [43] found that more screen time was associated with worse mental health, increased behavioral problems, decreased academic performance, and poorer sleep. These studies have consistently demonstrated the correlation between screen time and poorer physical health status. However, it is important to note that these studies were cross-sectional and cannot exclude reverse causality. Subsequent research should conduct more longitudinal cohort studies to ascertain the effects of excessive screen time. Nevertheless, we advocate for adolescents and their guardians to manage the screen time wisely.

The association between screen time and the risk of LBP may be due to bad postures. Forward bending/inclination of the back and head were often observed when using computer. Prolonged sitting with a smartphone may result in slumped posture among adolescents with LBP [44]. Smartphone use increased the flexion angles of the cervical and upper thoracic regions among university students [45]. Filho et al. [46] also found that slumping postures while watching TV and using computer were associated with chronic LBP among high school adolescents. LBP caused by bad postures may be related to the imbalance, weakness of muscles, and the stiffness of thoracolumbar fascia. Wong et al. [47] found that long-term hunched sitting decreased the activity of the internal oblique and transverse abdominal muscles. Fujitani et al. [48] found that recurrence of LBP significantly reduced the activity of posture control muscles. And Chen et al. [49] found that the thoracolumbar fascia stiffened significantly in a sitting position and increased with forward trunk positioning. According to previous research, correcting bad postures [50] and avoiding prolonged sitting or standing [51] are effective ways of preventing muscle fatigue and LBP, which should be widely publicized.

The study has several strengths. Our study assessed quantitatively the relationship between screen time and the risk of LBP by using a dose-response meta-analysis. And the linear relationship between daily computer use and LBP was found for the first time in our results. Further, this study included a large sample of children and adolescents, which enabled us to generalize our findings to the broader population. However, limitations should be cleared when interpreting the results. Firstly, there was no information on the classification, duration, and etiology of LBP in the studies included. Additionally, the risk of LBP may have been exaggerated because most studies reported total daily screen time without considering the level of physical activity during this period. And the participants’ daily screen time mainly was gathered from self-reported questionnaires, which were subject to memory bias. Lastly, there was no way to exclude reverse causality since all the included studies were cross-sectional.

## Conclusions

According to our results, screen time is positively related to the risk of LBP, and there is a linear relationship between daily computer use and the risk of LBP. Several strategic measures should be taken to prevent adolescents from developing severe LBP.

## Abbreviations

AHRQ: American Institute for Health Care Research and Quality Scale
CI: Confidence interval
COVID-19: Corona Virus Disease 2019
LBP: Low back pain
OR: odds ratio

## Declarations

### Availability of data and material

All the data included in this manuscript was available through the internet database.
